# Pleuropulmonary MDCT Findings: Comparison between Children with Pulmonary Vein Stenosis and Prematurity-Related Lung Disease

**DOI:** 10.3390/children9030355

**Published:** 2022-03-04

**Authors:** Abbey J. Winant, Sara O. Vargas, Kathy J. Jenkins, Ryan Callahan, Vanessa Rameh, Katie A. Krone, Patrick R. Johnston, Mirjam L. Keochakian, Edward Y. Lee

**Affiliations:** 1 Department of Radiology, Boston Children’s Hospital, Harvard Medical School, 300 Longwood Avenue, Boston, MA 02115, USA; abbey.winant@childrens.harvard.edu (A.J.W.); vanessa.rameh@childrens.harvard.edu (V.R.); patrick.johnston@childrens.harvard.edu (P.R.J.); 2 Department of Pathology, Boston Children’s Hospital, Harvard Medical School, 300 Longwood Avenue, Boston, MA 02115, USA; sara.vargas@childrens.harvard.edu; 3 Department of Cardiology, Boston Children’s Hospital, Harvard Medical School, 300 Longwood Avenue, Boston, MA 02115, USA; kathy.jenkins@childrens.harvard.edu (K.J.J.); ryan.callahan@childrens.harvard.edu (R.C.); mirjam.keochakian@childrens.harvard.edu (M.L.K.); 4 Division of Pulmonary Medicine, Boston Children’s Hospital, Harvard Medical School, 300 Longwood Avenue, Boston, MA 02115, USA; katie.krone@childrens.harvard.edu

**Keywords:** pulmonary vein stenosis, prematurity-related lung disease, multidetector computed tomography (MDCT), pleuropulmonary findings, children, pediatric patients

## Abstract

Purpose: To retrospectively compare the pleuropulmonary MDCT findings in children with pulmonary vein stenosis (PVS) and prematurity-related lung disease (PLD). Materials and Methods: All consecutive infants and young children (≤18 years old) who underwent thoracic MDCT studies from July 2004 to November 2021 were categorized into two groups—children with PVS (Group 1) and children with PLD without PVS (Group 2). Two pediatric radiologists independently evaluated thoracic MDCT studies for the presence of pleuropulmonary abnormalities as follows—(1) in the lung (ground-glass opacity (GGO), triangular/linear plaque-like opacity (TLO), consolidation, nodule, mass, cyst(s), interlobular septal thickening, and fibrosis); (2) in the airway (bronchial wall thickening and bronchiectasis); and (3) in the pleura (thickening, effusion, and pneumothorax). Interobserver agreement between the two reviewers was evaluated with the Kappa statistic. Results: There were a total of 103 pediatric patients (60 males (58.3%) and 43 females (41.7%); mean age, 1.7 years; range, 2 days–7 years). Among these 103 patients, 49 patients (47.6%) comprised Group 1 and the remaining 54 patients (52.4%) comprised Group 2. In Group 1, the observed pleuropulmonary MDCT abnormalities were—pleural thickening (44/49; 90%), GGO (39/49; 80%), septal thickening (39/49; 80%), consolidation (4/49; 8%), and pleural effusion (1/49; 2%). The pleuropulmonary MDCT abnormalities seen in Group 2 were—GGO (45/54; 83%), TLO (43/54; 80%), bronchial wall thickening (33/54; 61%), bronchiectasis (30/54; 56%), cyst(s) (5/54; 9%), pleural thickening (2/54; 4%), and pleural effusion (2/54; 4%). Septal thickening and pleural thickening were significantly more common in pediatric patients with PVS (Group 1) (*p* < 0.001). TLO, bronchial wall thickening, and bronchiectasis were significantly more frequent in pediatric patients with PLD without PVS (Group 2) (*p* < 0.001). There was high interobserver kappa agreement between the two independent reviewers for detecting pleuropulmonary abnormalities on thoracic MDCT angiography studies (k = 0.99). Conclusion: Pleuropulmonary abnormalities seen on thoracic MDCT can be helpful for distinguishing PVS from PLD in children. Specifically, the presence of septal thickening and pleural thickening raises the possibility of PVS, whereas the presence of TLO, bronchial wall thickening and bronchiectasis suggests PLD in the pediatric population.

## 1. Introduction

Pulmonary vein stenosis (PVS) is defined as a progressive reduction in luminal caliber of one or more large (extra-pulmonary) pulmonary veins [1,2,3,4]. In adults, PVS can occur as a complication of ablation for atrial fibrillation near the pulmonary veins [5,6]. In pediatric patients, PVS has different clinical associations. Pediatric PVS typically arises in infancy, and may be primary or secondary. Primary PVS arises in the absence of identifiable congenital heart disease, and is sometimes associated with prematurity [1,2,3,4,7]. Secondary PVS is more common, occurring after surgical repair of congenital heart disease (e.g., anomalous pulmonary venous connections or cor triatriatum), or may also arise “secondarily” in congenital cardiovascular anomalies that have not been operated upon, especially those with prolonged exposure to left to right shunts [8]. Both primary and secondary PVS are characterized by an abnormal proliferation of myofibroblast-like cells [9,10]. In both children and adults, non-invasive imaging, particularly multidetector CT (MDCT), which can visualize the decreased luminal caliber of the affected pulmonary vein(s), is important for the initial diagnosis and follow-up evaluation of PVS after treatment [11,12,13,14,15].

Recently, there has been an important paradigm shift in the imaging evaluation of pediatric PVS, change from solely focusing on the identification of affected pulmonary veins to also visualizing the characteristic extravascular thoracic findings of PVS in pediatric patients [16,17,18]. Identification of the characteristic extravascular thoracic findings of PVS in children is important because it can both confirm the diagnosis of PVS (when a narrowed pulmonary vein is visualized or suspected), and it can also suggest the diagnosis of PVS on MDCT studies that were not technically optimized for evaluation of the pulmonary veins. Furthermore, this new focus on “seeing the bigger picture” of PVS has improved our understanding of the pathophysiology of this elusive disease. In children with PVS, thoracic MDCT often visualizes a soft tissue proliferation along the pulmonary veins (likely corresponding to the myofibroblast-like proliferation) as well as interlobular septal thickening and alveolar ground-glass opacity (GGO) attributed to decreased pulmonary venous drainage (due to narrowed pulmonary veins) [16,17,18].

Despite several recently published studies demonstrating these characteristic extravascular MDCT abnormalities involving the lungs and pleura of pediatric patients with PVS, accurate diagnosis of PVS in pediatric patients continues to be challenging due to difficulties in distinguishing the pleuropulmonary MDCT abnormalities of PVS from prematurity-related lung disease (PLD), which is one of the main differential diagnostic considerations [7,19,20]. Early and accurate diagnosis of each of these conditions is critical to guiding appropriate treatment and management. To the best of our knowledge, there has been no prior study that directly compares the pleuropulmonary MDCT findings in children with PVS and PLD [1,4,21]. Therefore, the goal of our study was to retrospectively compare the pleuropulmonary MDCT findings in children with PVS and PLD.

## 2. Methods

### 2.1. Institutional Review Board Approval

Our institutional review board approved this retrospective review of patient’s medical records and MDCT studies. Due to the retrospective nature of this study, informed consent was waived. The study was conducted in accordance with the principles of the Declaration of Helsinki.

### 2.2. Patient Population

A computerized search of our hospital’s radiology, cardiology, pathology, and pulmonary department databases was used to identify consecutive pediatric patients (≤18 years old) who underwent thoracic MDCT studies from July 2004 to November 2021. Of note, the beginning date of July 2004 was selected because a new MDCT scanner (16-row MDCT) was installed in our hospital at that time. The resultant patient list was reviewed, and patients meeting criteria for Group 1 and Group 2 were included. Group 1 was composed of pediatric patients with PVS confirmed by echocardiogram and/or conventional angiography and without a history of prematurity. Group 2 was composed of pediatric patients with a history of prematurity and without PVS. In addition, for each patient, demographic information (e.g., age and gender) was also collected.

A total of 103 pediatric patients (60 males (58.3%) and 43 females (41.7%); mean age, 1.7 years; range, 2 days–7 years) met Group 1 or Group 2 criteria. Among these 103 patients, 49 patients (47.6%) belonged to Group 1 and the remaining 54 patients (52.4%) belonged to Group 2. For each patient, only the initial thoracic MDCT study performed at the time of diagnosis was included. Therefore, there were 49 thoracic MDCT studies from 49 individual pediatric patients with PVS (Group 1) and there were 54 thoracic MDCT studies from 54 individual pediatric patients with PLD (Group 2).

### 2.3. Prematurity and Pulmonary Vein Stenosis Diagnostic Criteria

Prematurity was defined as born alive before 37 weeks of pregnancy [22]. The PVS diagnostic criterion was pulmonary vein luminal narrowing in ≥2 vessels with a mean gradient ≥ 4 mm Hg seen on echocardiography or conventional angiography [16,17,18].

### 2.4. Thoracic MDCT Technical Factors

#### 2.4.1. Types of MDCT Scanners

For Group 1, six different MDCT scanners were used for 49 thoracic MDCT studies in the final study group including: (1) a 16-MDCT scanner (n = 7; 14%); (2) a 64-MDCT scanner (n = 21; 43%); (3) a 96-MDCT scanner (n = 17; 35%); (4) a 128-MDCT scanner (n = 1; 2%); (5) a 256-MDCT scanner (n = 2; 4%); and (6) a 302-MDCT scanner (n = 1; 5%).

For Group 2, four different MDCT scanners were used for 54 thoracic MDCT studies in the final study group including: (1) a 16-MDCT scanner (n = 1; 2%); (2) a 40-MDCT scanner (n = 2; 3%); (3) a 64-MDCT scanner (n = 9; 17%); and (4) a 96-MDCT scanner (n = 42; 78%).

#### 2.4.2. Thoracic MDCT Technical Parameters

Low-radiation-dose MDCT techniques using tube current with dose modulation, weight-based kilovoltage, and a high-speed mode (rotation time ≤ 1 s) were used based on the ALARA (As Low As Reasonably Achievable) principle for the thoracic MDCT studies included in both Group 1 and Group 2. In Group 1, all 49 thoracic MDCT studies were obtained with intravenous contrast with the dose of 1.5–2 mL/kg. In Group 2, 33 (61%) thoracic MDCT studies were obtained with intravenous contrast while the remaining 29 (39%) thoracic MDCT studies were performed without intravenous contrast. The entire chest was imaged from the level of the thoracic inlet to the level of the diaphragm in the cranial-to-caudal direction.

### 2.5. Thoracic MDCT Image Review

The axial CT data set was obtained and subsequently reconstructed into two-dimensional coronal and sagittal reformatted MDCT image reconstructions in standard lung (level, −500 Hounsfield units (HU); width, 1500 HU) and soft tissue (level, 40 HU; width, 450 HU) window settings at the CT console for review. The reviewers used a PACS (picture archiving and communication system) (Synapse, Fujifilm Medical Systems, Stamford, CT, USA) for MDCT image review.

In order to eliminate potential reviewer bias, patient identifiers were removed from the thoracic MDCT studies and all thoracic MDCT studies were randomized prior to reviewing. In addition, the two reviewers were blinded to the clinical history as well as the reports of the current and any prior imaging studies.

Two pediatric radiologists (a pediatric thoracic radiologist and a pediatric radiology fellow with 11 and 5 years of experience in interpreting pediatric thoracic MDCT studies, respectively) independently reviewed all thoracic MDCT studies. For discrepant cases between two reviewers, a third radiologist, a pediatric thoracic radiologist with more than 20 years of experience in interpreting thoracic MDCT studies, served as an arbitrator, and made the final decision. In order to prevent potential bias, this third reviewer was blinded to the two initial reviewer’s decisions on disagreed cases and all other clinical and imaging study information.

### 2.6. Thoracic MDCT Study Image Assessment

The lung, airway, and pleura were systemically evaluated based on previously established criteria [23,24].

The lung was evaluated for the presence of: (1) ground-glass opacity (GGO); (2) triangular/linear plaque-like opacity (TLO); (3) consolidation; (4) nodule; (5) mass; (6) cyst(s); (7) septal thickening; and (8) fibrosis. GGO was defined as an area of hazy increased lung opacity with indistinct margins of pulmonary vessels. The diagnosis of TLO was made when there was relatively thick triangular-shaped or linear plaque-like opacity. Consolidation was defined as a homogeneous increase in pulmonary parenchyma attenuation, which obscures the margins of adjacent vessels and airway walls. The diagnosis of nodule was made when a rounded or irregular opacity measuring up to 3 cm in diameter was seen. A mass was defined as a solid or partly solid opacity that was larger than 3 cm in diameter. The diagnosis of cyst(s) was made when a round parenchymal lucency or low-attenuating area with a well-defined interface with normal lungs was visualized. Septal thickening was defined as a prominent thin linear opacity along the interlobular septum at right angles to and in contact with the pleural surfaces. Fibrosis was diagnosed when there were reticular opacities and honeycombing (closely approximated ring shadows typically of 3–10 mm in diameter with walls of 1–3 mm in thickness, resembling a honeycomb).

The airway was evaluated for the presence of: (1) bronchial wall thickening and (2) bronchiectasis. The bronchial wall thickening was defined as the degree to which the bronchial wall was thicker than the wall of the adjacent pulmonary vessels. The diagnosis of bronchiectasis was made when there was bronchial dilatation with respect to the accompanying pulmonary artery (signet ring sign), a lack of tapering of bronchi, and identification of bronchi within 1 cm of the pleural surface.

The pleura were evaluated for the presence of: (1) pleural thickening; (2) pleural effusion; and (3) pneumothorax. Pleural thickening was defined as abnormally increased thickness of the lining of the pleura. The diagnosis of pleural effusion was made when there was fluid within the pleural cavity. A pneumothorax was diagnosed when the pleural space contained air.

### 2.7. Statistical Analysis

Continuous variables were summarized via means, standard deviations, and ranges. The number and percentage of abnormalities were calculated based on the proportions of abnormalities detected in thoracic MDCT studies. For each type of abnormality, Group 1 (with PVS) and Group 2 (with PLD) were compared with respect to the proportion of abnormalities using Fisher’s exact test. Interobserver agreement between the two independent reviewers regarding thoracic MDCT findings was measured by the Kappa statistic. As a guide for interpreting magnitudes for Kappa, we defined low, moderate, and high agreement via the ranges 0.5–0.75, 0.75–0.9, and 0.9–1, respectively. Fisher’s exact tests and Kappa estimates were performed using SAS/STAT^®^ 14.1 using the frequency analysis procedure (PROC FREQ) [25].

## 3. Results

### 3.1. Patient Population Characterization

Group 1 (with PVS) consisted of 49 consecutive pediatric patients (47.6%). There were 28 (57.1%) male and 21 (42.9%) female patients (mean age: 1.8 years; SD: 1.9 years; range: 3 days to 7 years). The gestational age of all patients included in this group was full term. Presented clinical signs and symptoms in these 49 pediatric patients included: failure to thrive (n = 8; 16.3%), pulmonary hypertension (n = 7; 14.3%), shortness of breath (n = 5; 10.2%), hypoxemia (n = 5; 10.2%), fever (n = 3; 6.1%), and bradycardia (n = 2; 4.1%). Extrapulmonary MDCT findings for a subset of these patients were previously reported [16,17,18].

Group 2 (with PLD) consisted of 54 consecutive pediatric patients (52.4%). There were 32 (59.3%) male and 22 (40.7%) female patients (mean age: 1.7 years; SD: 2.0 years; range: 2 days to 8 years). The gestational age of all patients included in this group was less than 37 weeks of pregnancy (mean gestation age = 28.3 weeks; SD = 2.9 weeks; range = 23–35 weeks). All patients included in this group lacked PVS. Presented clinical signs and symptoms in these 54 pediatric patients included: shortness of breath (n = 37; 68.5%), hypoxemia (n = 30; 55.6%), pulmonary hypertension (n = 13; 24%), failure to thrive (n = 13; 24%), and fever (n = 7; 13%).

### 3.2. Thoracic MDCT Findings

The summary of pleuropulmonary findings on thoracic MDCT studies in Group 1 (with PVS) and Group 2 (with PLD) is listed in Table 1.

### 3.3. Lung Findings

**Figure 1:**
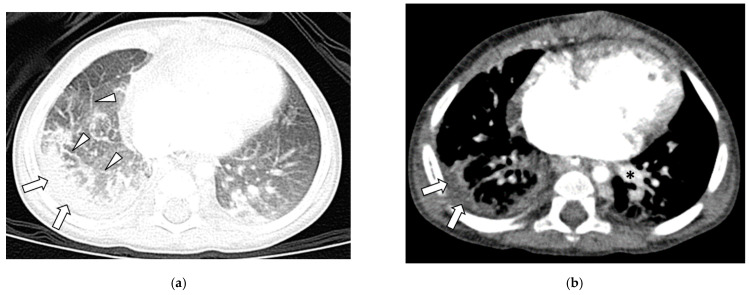
Fourteen-month-old girl with right sided pulmonary vein stenosis. (**a**): Axial lung window CT image demonstrates septal thickening (arrowheads) and pleural thickening (arrows) in the right hemithorax. (**b**): Axial contrast-enhanced soft tissue window CT image shows pleural thickening (arrows) in the right hemithorax. Right pulmonary veins are absent. Left pulmonary vein (asterisk) is visualized.

**Figure 2:**
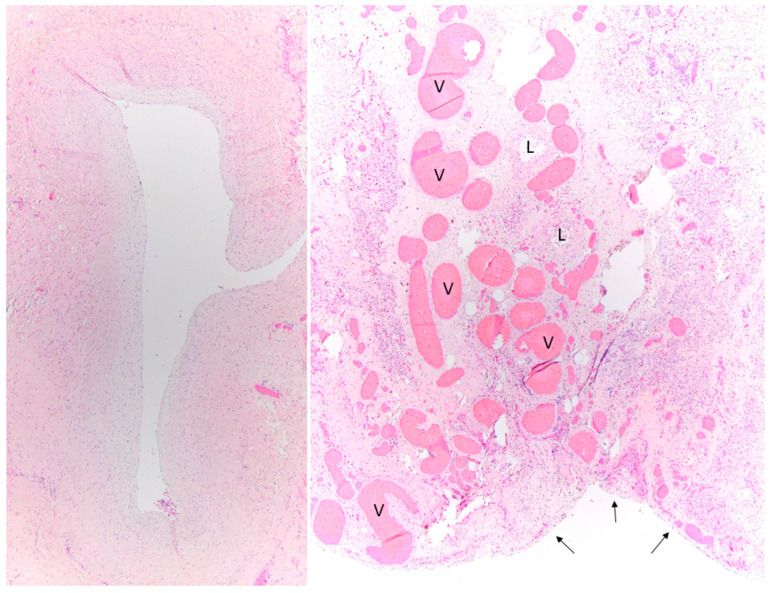
Histologic findings in pulmonary vein stenosis. After death at age 13 months, this patient’s pulmonary veins showed fibrous intimal proliferation (left panel; hematoxylin and eosin; original magnification, 40×). The pleura (arrows) and interlobular septum were thickened, containing distended and tortuous veins (V) and thick-walled lymphatic channels (L) (right panel; hematoxylin and eosin; original magnification, 100×).

In Group 1 (with PVS), among 49 thoracic MDCT studies, two lung abnormalities were observed including GGO in 39 studies (76%) and septal thickening in 39 studies (76%) (Figure 1a and Figure 2).

**Figure 3:**
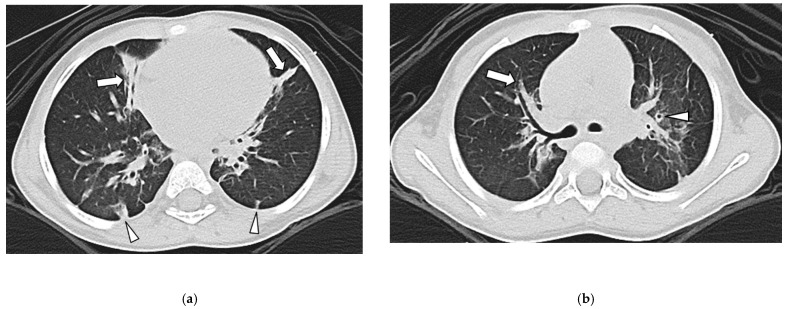
Three-year-old girl with prematurity-related lung disease. (**a**): Axial lung window CT image shows triangular/linear plaque-like opacity (arrows) in both lungs. Pleural based atelectasis (arrowheads) is also seen. However, no pleural thickening is noted. (**b**): Axial lung window CT image demonstrates bronchial wall thickening (arrowhead) and bronchiectasis (arrow).

**Figure 4:**
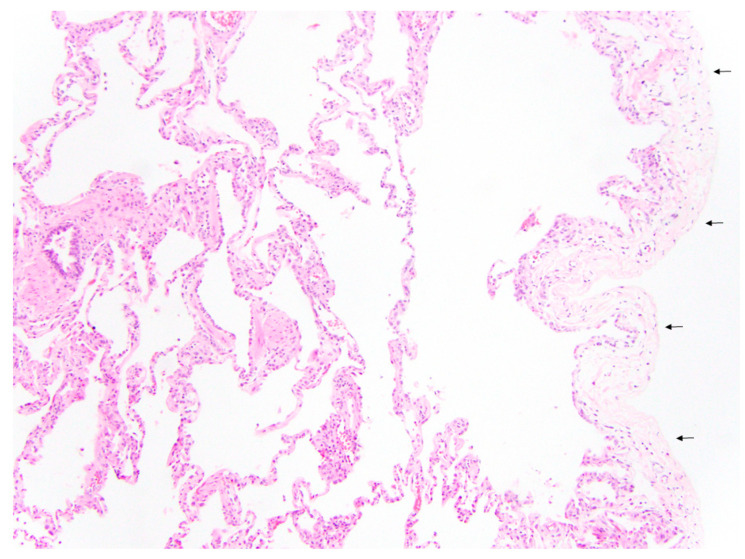
Histology in prematurity-related lung disease. Wedge biopsy sample from a 2-year-old girl born at 32 and 5/7 weeks’ gestational age shows small airway remodeling, smooth muscle proliferation, and enlarged airspaces. The pleura (arrows) were thin and delicate without pathological evidence for thickening. (Hematoxylin and eosin; original magnification, 100×).

In Group 2 (with PLD), among 54 MDCT studies, five lung abnormalities were observed including GGO in 45 studies (83%), TOL in 43 studies (80%), consolidation in 8 studies (15%), and cyst(s) in 5 studies (9%) (Figure 3a and Figure 4).

The presence of septal thickening was statistically significantly more frequently observed in Group 1 (with PVS) (80% vs. 0%, *p* ≤ 0.001), whereas the presence of TOL was statistically significantly more frequently found in Group 2 (0% vs. 80%, *p* < 0.001).

### 3.4. Airway Findings

In Group 1 (with PVS), in 49 thoracic MDCT studies, no bronchial wall thickening or bronchiectasis were observed. In Group 2 (with PLD), in 54 thoracic MDCT studies, bronchial wall thickening was seen in 33 studies (61%) and bronchiectasis in 30 studies (56%) (Figure 3b). Therefore, the presence of bronchial wall thickening and bronchiectasis were statistically significantly more frequently found in Group 2 (with PLD) (0% vs. 61%, *p* < 0.001).

### 3.5. Pleural Findings

In Group 1 (with PVS), in 49 thoracic MDCT studies, pleural thickening was seen in 44 studies (90%) (Figure 1b). In Group 2 (with PLD), in 54 thoracic MDCT studies, pleural thickening was seen in two studies (4%) and pleural effusion in two studies (4%). No pneumothorax was seen in both Group 1 and Group 2. The presence of pleural thickening was statistically significantly more frequently in Group 1 (with PVS) (90% vs. 4%, *p* < 0.001).

### 3.6. Interobserver Agreement

The initial two reviewers were in agreement on all pleuropulmonary MDCT findings among all categories, with the exception of six occasions on the 103 included thoracic MDCT studies. The disagreed incidences between the two initial reviewers were related to the presence of bronchiectasis in one case, septal thickening in three cases, and bronchial wall thickening in three cases. The third reviewer, the tie-breaker, decided that bronchiectasis was present in one case, septal thickening was present in two cases, and bronchial wall thickening was present in all three cases. There was a high interobserver kappa agreement between the two initial reviewers for diagnosing pleuropulmonary abnormalities detected on thoracic MDCT studies. The proportion of agreement was 0.99 with a 95% CI (confidence interval) of 0.992 and 0.999, and the Kappa statistic was 0.99 with a 95% CI of 0.98 and 1.00.

## 4. Discussion

The results of our study showed that the pleuropulmonary abnormalities detected on thoracic MDCT are significantly different in pediatric patients with PVS versus those with PLD. Septal thickening and pleural thickening were significantly more frequently seen in pediatric patients with PVS. In contrast, TLO, bronchial wall thickening, and bronchiectasis were significantly more frequently seen in pediatric patients with PLD without PVS. Therefore, the findings of our study demonstrate a clinically important difference in the thoracic MDCT imaging findings of PVS and PLD that can be helpful for distinguishing pediatric patients with PVS from PLD in daily clinical practice.

We believe that the pleuropulmonary MDCT findings observed in both groups (PVS and PLD) can be explained by the pathophysiology of each disorder. First, the septal thickening and pleural thickening that were 100% specific to pediatric patients with PVS most likely reflect the elevated pulmonary venous pressure in the interlobular septa and visceral pleural surface, resulting from the obstruction of pulmonary venous return due to narrowed pulmonary veins. In contrast, the TLO that was 100% specific to pediatric patients with PLD likely reflects a combination of underlying lung parenchymal architectural distortion, atelectasis, and fibrous scarring. In addition, we postulated that the bronchial wall thickening and bronchiectasis that were 100% specific for patients with PLD may be due to the chronic inflammatory remodeling of airways following hyaline membrane disease and its therapy, accompanied by limited or decreased clearance of mucus and recurrent superimposed infections in the underdeveloped immature lungs and airways.

Interestingly, in both groups (PVS and PLD), GGO was frequently seen, with GGO observed in up to 76% of pediatric patients with PVS and 83% of pediatric patients with PLD. We believe that the pathophysiologic mechanism behind the development of GGO is different in each of these conditions. For example, we suspect that the GGO seen in pediatric patients with PVS may reflect the consequences of elevated pulmonary venous pressure, including intra-alveolar filling due to macrophages, edema fluid, and hemosiderosis and alveolar septal thickening due to distended capillaries. In contrast, variable involvement by micro-atelectasis, alveolar septal fibrosis, smooth muscle proliferation, superimposed infectious processes, or a combination thereof may have contributed to the thoracic MDCT finding of GGO in pediatric patients with PLD. In addition, since GGO was frequently seen at similar rates (76% in PVS and 83% in PLD) in both groups (PVS and PLD), we believe that this particular thoracic MDCT finding (GGO) cannot be used to differentiate PVS from PLD in the pediatric population.

The findings of our study correlate well with the findings from previously published studies in pediatric patients with primary and secondary PVS focusing on extrathoracic MDCT findings [16,17,18]. These studies also identified the presence of three main extravascular pleuropulmonary abnormalities on thoracic MDCT in pediatric patients with PVS: GGO, septal thickening, and pleural thickening. The frequencies of these thoracic MDCT abnormalities were 93–95% for GGO, 33–45% for septal thickening, and 80–93% for pleural thickening, similar to the findings seen in our study [16,17,18]. Importantly, our study extends previous findings by demonstrating that in a case-control cohort of patients with PVS and PLD, these MDCT abnormalities are exclusive to PVS.

Regarding pediatric patients with PLD, the TLO, bronchial wall thickening, and bronchiectasis have been typically observed in pediatric patients with PLD in thoracic MDCT studies [19,26,27]. However, the presence of consolidation (15%) and cyst(s) (9%) seen in pediatric patients with PLD in our study has not been observed in the previously published studies. We believe that this difference is most likely related to the difference in patient population. More specifically, the clinical indication for thoracic CT studies in pediatric patients with PLD (group 2) was often for the investigation of possible lung infection. Therefore, we believe that the consolidation seen in our study was likely due to superimposed infectious processes, and the cysts may reflect lung abscesses, post-infectious pneumatoceles, or iatrogenic pneumatoceles secondary to prior pulmonary interstitial emphysema in the setting of positive-pressure ventilation on poorly compliant immature lungs. Furthermore, our finding that TLO, bronchial thickening, and bronchiectasis had 100% specificity for PLD in this study cohort comparing PVS and PLD is novel. The highly specific nature (100%) of septal thickening and pleural thickening for PVS and of TLO, bronchial thickening, and bronchiectasis for PLD support a reliable utility for these features in settings where the clinical differential diagnosis for pediatric lung disease includes PVS and PDL. While patients with co-existing PVS and PDL were not included in this study, our results suggest that in patients with features specific to PDL (e.g., TLO and/or bronchial wall thickening) who also show features specific to PVS (e.g., thickened pleura and/or thickened interlobular septa), the diagnosis of co-existing PVS/PDL should be considered.

There are three main potential limitations in our study. First, various thoracic MDCT techniques (i.e., intravenous (IV) contrast administration, types of MDCT scanners, and slice thickness) were used in our study population mainly due to the clinical indication-based thoracic MDCT protocol used in our department and the advances in MDCT scanners that have occurred over the years. However, we believe that these variables most likely did not affect the results of our study substantially because the administration of IV contrast does not affect the evaluation of lungs and all CT images were obtained with a thin-section technique (<1 mm). Secondly, the population size was relatively small in both groups. However, it is important to emphasize that the small study population was mainly due to the rarity of disorder (i.e., PVS) and MDCT is not currently routinely used for evaluating PLD in the pediatric population unless there is a specific clinical indication, such as the evaluation of a superimposed infectious process. A future multicenter study, which can provide a larger patient population, will be helpful for confirming the results of our study. Lastly, not all patients included in this study had pathological confirmation of thoracic MDCT findings. However, it is worth emphasizing that pathological confirmation is not currently routinely performed for evaluation of pleuropulmonary abnormalities in pediatric patients with known PVS and PLD.

In conclusion, our study is the first study to directly compare the pleuropulmonary MDCT findings in children with PVS and PLD. We believe that our findings of significantly different thoracic MDCT pleuropulmonary abnormalities between children with PVS and children with PLD provide clinically valuable new information that can be used to distinguish pediatric patients with PVS from PLD in daily clinical practice. Recognition of these distinct characteristic pleuropulmonary abnormalities (i.e., septal and pleural thickening in PVS versus TLO, and bronchial thickening and bronchiectasis in PLD) can allow early and accurate diagnosis of these two important disease processes. Therefore, our findings of statistically significant differences in the characteristic pleuropulmonary MDCT abnormalities of PVD and PLD have great potential to improve diagnostic accuracy for these two pediatric thoracic disorders.

## Figures and Tables

**Table 1:** Summary of pleuropulmonary thoracic MDCT findings of children with pulmonary vein stenosis (PVS) versus prematurity-related lung disease (PLD).

Types of Pleuropulmonary Thoracic MDCT Findings	Group 1 (PVS) Number (Percentage) of Abnormalities (n = 49)	Group 2 (PLD) Number (Percentage) of Abnormalities (n = 54)	*p* Value
Lung Findings			
GGO	39/49 (76%)	45/54 (83%)	0.800
TOL	0/49 (0%)	43/54 (80%)	<0.001
Consolidation	0/49 (0%)	8/54 (15%)	0.531
Nodule	0/49 (0%)	0/54 (0%)	1.000
Mass	0/49 (0%)	0/54 (0%)	1.000
Cyst(s)	0/49 (0%)	5/54 (9%)	0.058
Septal Thickening	39/49 (76%)	0/54 (0%)	<0.001
Fibrosis	0/49 (90%)	0/54 (0%)	1.00
Airway Findings			
Bronchial Wall Thickening	0/49 (0%)	33/54 (61%)	<0.001
Bronchiectasis	0/49 (0%)	30/54 (56%)	<0.001
Pleural Findings			
Pleural Thickening	44/49 (90%)	2/54 (4%)	<0.001
Pleural Effusion	0/49 (0%)	2/54 (4%)	1.000
Pneumothorax	0/49 (0%)	0/54 (0%)	1.000

MDCT: multidetector computed tomography; PVS: pulmonary vein stenosis; PLD: prematurity-related lung disease; n: number; GGO: ground-glass opacity; TOL: triangular/linear plaque-like opacity.
